# The effect of SGLT2 inhibitors, GLP-1 receptor agonists, or DPP-4 inhibitors on stroke risk in type 2 diabetes: a nationwide longitudinal cohort study in Sweden with contextualisation analysis

**DOI:** 10.1016/j.eclinm.2026.104220

**Published:** 2026-09-23

**Authors:** Anastasios Mavridis, Tamar Abzhandadze, Dongni Buvarp, Koen Simons, Björn Eliasson, Mia von Euler, Adam Viktorisson, Katharina S. Sunnerhagen

**Affiliations:** a Department of Clinical Neuroscience, Institute of Neuroscience and Physiology, Sahlgrenska Academy, University of Gothenburg, Sweden; b Department of Occupational Therapy and Physiotherapy, Sahlgrenska University Hospital, Gothenburg, Sweden; c Department of Neurology, Sahlgrenska University Hospital, Gothenburg, Sweden; d The Sahlgrenska University Hospital, Gothenburg, Sweden; e Biostatistics, School of Public Health and Community Medicine, Institute of Medicine, Sahlgrenska Academy, University of Gothenburg, Gothenburg, Sweden; f The National Diabetes Register, Center of Registers, Gothenburg, Sweden; g Sophiahemmet University, Stockholm, Sweden

**Keywords:** Cerebral infarction, Diabetes mellitus, Type 2, Glucagon-like peptide-1 receptor agonists, Sodium-glucose transporter 2 inhibitors, Dipeptidyl-peptidase IV inhibitors

## Abstract

**Background:**

The impact of SGLT2 inhibitors (SGLT2i), GLP-1 receptor agonists (GLP-1RA), and DPP-4 inhibitors (DPP-4i) on stroke risk in individuals with type 2 diabetes remains uncertain. Meta-analyses of randomised controlled trials (RCTs) show a protective effect for GLP-1RA but neutral findings for SGLT2i and DPP-4i, whereas observational studies often suggest broader stroke risk reductions. This study aimed to estimate the associations of SGLT2i, GLP-1RA, and DPP-4i with stroke risk in nationwide longitudinal data, and to contextualize these results within existing meta-analyses of RCTs and observational studies.

**Methods:**

This was a nationwide longitudinal study including individuals with type 2 diabetes first registered in the Swedish National Diabetes Register between January 1, 2013, and December 31, 2019. Individuals with a prior stroke or insufficient data for multiple imputation were excluded. The primary outcome was ischemic stroke. Treatments, comorbidities, and laboratory values were updated over time. Marginal structural models with inverse probability of treatment weighting and weighted Cox regression were used to estimate hazard ratios (HRs) for ischemic, hemorrhagic and total stroke pooled across five multiply imputed datasets. To contextualise the findings, PubMed was searched from inception to June 23, 2026, for meta-analyses of randomised controlled trials and observational studies reporting stroke outcomes for the three drug classes.

**Findings:**

We included 171,917 individuals with type 2 diabetes [59.2% males, baseline mean (SD) age 62.7 (11.6), mean (SD) follow-up 3.0 (1.9) years] contributing 1,617,462 clinical registrations. During follow-up, 2734 (1.59%) ischemic and 428 (0.25%) hemorrhagic strokes occurred. GLP-1RA use was associated with a lower risk of ischemic (HR 0.46, 95% CI 0.29–0.72) and total stroke (HR 0.51, 95% CI 0.33–0.79), whereas no clear associations were observed for hemorrhagic stroke. SGLT2i and DPP-4i showed no clear associations with any stroke outcome. The contextualisation identified 91 meta-analyses. RCT meta-analyses generally showed a protective effect for GLP-1RA and neutral effects for SGLT2i and DPP-4i, while observational meta-analyses suggested protective associations for GLP-1RA and SGLT2i.

**Interpretation:**

In this nationwide study, GLP-1RA were associated with reduced stroke risk, while SGLT2i and DPP-4i showed neutral effects. The overall direction of findings was consistent with evidence from RCT meta-analyses. Future research should investigate how these therapies can be optimally incorporated into treatment strategies for stroke prevention in individuals with type 2 diabetes.

**Funding:**

Sahlgrenska University Hospital's funds, the Swedish National Stroke Association, the Swedish Brain Foundation, the Swedish Heart Lung Foundation, the Bygg-Göta Foundation for scientific research, the Winberg foundation, and the Swedish state under the ALF agreement between the Swedish government and the county councils.

**Research in context**

**Evidence before this study**

We searched PubMed from inception to June 23, 2026, for meta-analyses of randomised controlled trials (RCTs) and observational studies using the terms (sodium-glucose cotransporter 2 OR SGLT2 OR SGLT-2 OR GLP1 OR GLP-1 OR glucagon-like peptide-1 OR DPP4 OR DPP-4 OR dipeptidyl peptidase-4) AND (stroke OR “cerebral infarction” OR “intracerebral hemorrhage”) AND diabetes, filtered for Meta-Analyses. Of 274 abstracts and 102 full texts screened, meta-analyses of RCTs for SGLT2 inhibitors consistently showed no effect on stroke risk (effect sizes 0.84–1.30), whereas observational meta-analyses suggested a protective association (0.75–0.87). For GLP-1 receptor agonists, both RCTs and observational studies showed a consistent protective effect (0.80–0.91). For DPP-4 inhibitors, RCTs indicated a neutral effect (0.77–1.03), and only one meta-analysis including observational data was identified, which also reported a neutral effect. Observational studies that consider time-varying treatment patterns and confounding in routine clinical data may help contextualize differences between trial and real-world findings.

**Added value of this study**

This was a nationwide, real-world longitudinal study using data from the Swedish National Diabetes Register, including 171,917 individuals with up to 7 years of follow-up and 1,617,462 clinical registrations. We applied inverse probability of treatment weighting to address time-varying confounding, treatment switching, and treatment–confounder feedback. To our knowledge, this is one of the largest nationwide register-based studies to examine stroke risk with modern glucose-lowering therapies under routine clinical use. Treatment use and clinical characteristics were updated throughout follow-up to reflect routine clinical practice. GLP-1RA use was associated with a lower risk of ischemic stroke (HR 0.46, 95% CI 0.29–0.72) and total stroke (HR 0.51, 95% CI 0.33–0.79), whereas no clear associations were observed for hemorrhagic stroke. SGLT2i and DPP-4i showed no clear associations with ischemic, hemorrhagic, or total stroke.

**Implications of all the available evidence**

Our findings together with existing meta-analyses support a protective association between GLP-1RA use and stroke risk, while showing no clear association for SGLT2i or DPP-4i. By accounting for evolving treatment use, treatment switching, and time-varying confounding, this study extends existing evidence by characterizing stroke risk under the dynamic treatment patterns encountered in routine clinical practice. Future research should determine how these findings can be translated into personalised glucose-lowering strategies that optimise stroke prevention in type 2 diabetes.

## Introduction

Type 2 diabetes increases the risk of ischemic stroke,^1^^,^^2^ a leading global cause of death and disability.^3^ Sodium-glucose cotransporter-2 (SGLT2i) inhibitors, glucagon-like peptide-1 receptor agonists (GLP-1RA), and dipeptidyl peptidase-4 (DPP-4i) inhibitors are widely used in routine care for glycaemic control.^4^ Growing evidence also suggests additional cardiovascular benefits.^5^ However, the importance for stroke prevention remains uncertain, with discordance between randomised controlled trials (RCTs) and observational data.^6^ Meta-analyses of placebo-controlled RCTs consistently suggest a 2%–23% reduction in stroke risk with GLP-1RA, while neutral effects are found for SGLT2i and DPP-4i.^7^, ^8^, ^9^ Several real-world data studies, however, fail to identify a significant difference between SGLT2i and GLP-1RA,^6^^,^^10^^,^^11^ while others have associated SGLT2i,^12^, ^13^, ^14^, ^15^ and DPP-4i^16^^,^^17^ with a protective effect against stroke. Identifying which glucose-lowering agents confer protection against stroke is crucial for clinical decision-making and public health policy.

Randomised controlled trials provide strong evidence on the cardiovascular effects of glucose-lowering therapies, including stroke outcomes.^7^, ^8^, ^9^ At the same time, observational studies describing treatment use in routine clinical practice have reported heterogeneous findings.^6^^,^^10^, ^11^, ^12^, ^13^, ^14^, ^15^, ^16^, ^17^ Such differences may reflect variations in study populations, follow-up, and design-related features of observational studies, including how treatment exposure and clinical characteristics evolve over time in real-world settings.^18^ We hypothesized that consideration of these longitudinal aspects would provide additional insight into previously reported differences in stroke risk estimates for GLP-1RA, SGLT2i, and DPP-4i.

This study aimed to estimate the effects of SGLT2i, GLP-1RA, and DPP-4i on ischemic, hemorrhagic and total stroke in individuals with type 2 diabetes and to contextualize these with findings from existing meta-analyses of randomised trials and observational studies.

## Methods

### Ethics

The study was conducted in accordance with the Declaration of Helsinki and was approved by the Swedish Ethical Review Authority on August 18th, 2021 (registration number: 2021–03645). Individual consent is not required to report patients to national quality registries of health care according to Swedish law (Patient Data Act 2008:355, chapter 7).

### Study design and data sources

This was a nationwide longitudinal study using real-world data from the Swedish National Diabetes Register (NDR). The NDR collects clinical and laboratory information on most individuals with diabetes in Sweden.^19^ Data are reported by participating healthcare units as part of routine diabetes care, either through automatic transfer from electronic health records or manual reporting, depending on local practice. Throughout this manuscript, each NDR entry is referred to as a clinical registration. In 2019 its coverage was estimated to 87%.^20^ Comorbidities and outcomes were collected from the National Patient Register, which includes information on hospital admissions and outpatient visits nationwide.^21^ Stroke outcomes were also collected from the National Stroke Register (RIKSSTROKE),^22^ which provides data on stroke events, treatments, and outcomes with an estimated coverage of 89% for 2019.^23^ Information on dispensed prescribed medications was collected from the National Prescribed Drug Register.^24^^,^^25^ Data linkage was performed by Statistics Sweden and the National Board of Health and Welfare using the personal identity numbers that are provided to all residents in Sweden at birth or shortly after immigration, and pseudonymized data were given to the researchers.

### Participants

Individuals with type 2 diabetes who were registered in the NDR for the first time between January 1st, 2013, and December 31st, 2019, were included in the study. The first NDR registration criterion was used to provide a clearly defined and comparable cohort-entry point across participants, from which subsequent treatment patterns and repeated clinical measurements could be followed longitudinally. The study period was selected because complete linked data were available for the study and to restrict follow-up to the pre–COVID-19 period, thereby avoiding potential changes in healthcare utilisation and outcomes during the pandemic. The exclusion criteria encompassed a prior diagnosis of stroke, and insufficient data (see missing data section). Intervals with no observable follow-up time after construction of the time-varying dataset were also removed (e.g., when the outcome occurred on the date of the first registration).

### Procedures

The NDR includes individuals with both Type 1 and Type 2 Diabetes. Diabetes type was established using epidemiological criteria^1^; Individuals diagnosed at any age and managed with dietary modifications alone, or with oral glucose-lowering medications, or insulin (with or without oral agents) if diagnosed at age 40 or older, were classified as type 2 diabetes. In the Swedish healthcare system, individuals with type 2 diabetes are generally managed in primary care.

### Variables

Sociodemographic characteristics included baseline age, sex, smoking status, and physical activity level (frequency of ≥30 min/week of an activity comparable to outdoor walking: Level 1, never; Level 2, <1 time/week; Level 3, 1–2 times/week; Level 4, 3–5 times/week; Level 5, 6–7 times/week).

Comorbidities were considered present continuously from the date of the diagnosis and onwards. The included conditions were cancer (ICD-10: C00–C97), dementia (F01–F09), alcohol or substance abuse (F10–F19), hypertension (I10–I15), atrial fibrillation (I48), ischemic heart disease (I20–I25), heart failure (I50, I110), respiratory conditions (J40–J47, J60–J70), vascular conditions (I70–I79, R02).

In Sweden, all prescribed medications are dispensed exclusively through community pharmacies, with each dispensation automatically recorded in the nationwide Prescribed Drug Register. Estimated intervals of medication use were calculated based on filled prescriptions, package information, and defined daily doses, with active medications continuously updated throughout the study period to account for drug switches (see Supplementary Methods). The included medication categories were SGLT2i, GLP-1RA, DPP-4i, insulin, other antidiabetic treatments, anticoagulants, antiplatelets, antihypertensives, lipid-lowering agents, diuretics and β-blockers/antiarrhythmics/glycosides. For each drug class of interest (SGLT2i, GLP-1RA, DPP-4i), exposure was treated as a time-varying variable and updated throughout follow-up. Treatment initiation or discontinuation were incorporated dynamically as they occurred in clinical practice, rather than treated as censoring events.

The included laboratory values and clinical measurements were HbA1c, Low Density Lipoprotein (LDL), Glomerular Filtration Rate (GFR), Systolic Blood Pressure (BP), Body Mass Index (BMI), and albuminuria (categorized as No/Normoalbuminuria, Microalbuminuria, Macroalbuminuria).

### Outcomes

The primary outcome was time from the first registration in the NDR to the first ischemic stroke (fatal or non-fatal), while the secondary outcomes were hemorrhagic and total stroke. Ischemic stroke was defined as ICD-10 code I63, hemorrhagic stroke as I61, while unspecified stroke (I64) was classified as ischemic, as Swedish stroke guidelines recommend immediate CT imaging for suspected stroke, and CT was performed in 98% of registered stroke cases,^23^ making it unlikely that hemorrhagic strokes would remain unspecified. For each outcome, follow-up began at the first NDR registration and continued until the first event of interest, death, a competing stroke subtype, or administrative censoring on 31 December 2019, whichever occurred first. For the ischemic stroke analysis, hemorrhagic stroke was treated as a censoring event, and vice versa for the hemorrhagic stroke analysis. Total stroke included the first occurrence of either ischemic or hemorrhagic stroke.

### Statistical analysis

Descriptive characteristics of the study sample were presented at baseline and longitudinally, using counts and valid percentages for categorical variables, and mean (SD) or median (IQR) for continuous variables. Longitudinal descriptives were calculated as the mean of individual-level means for continuous variables and as the proportion of individuals ever exposed for categorical variables.

The variables HbA1c, systolic blood pressure, BMI, LDL cholesterol, GFR, physical activity, and smoking status were imputed. Individuals, along with all their registrations, were excluded if (i) all of these variables were missing in at least one registration, or (ii) any one of these variables was missing in all registrations, as these patterns were considered insufficiently informative for reliable imputation.

Multiple imputation was performed using two-level predictive mean matching^26^ with five imputations and ten iterations. The imputation model accounted for the longitudinal structure of the data by clustering observations within individuals and including random effects for time. All covariates included in the time-updated treatment assignment models, including treatment and clinical variables, were incorporated into the imputation models. Stroke outcomes were not included. The procedure converged successfully, with convergence confirmed through trace plots (Supplementary Figure S1), and imputed and original values showing good agreement in the density plots (Supplementary Figure S2).

Because treatment and clinical characteristics changed over time, and previous treatment could influence subsequent clinical characteristics that in turn affected later treatment decisions (treatment–confounder feedback),^27^ IPTW within a marginal structural model (MSM) framework^28^^,^^29^ was used to account for time-varying confounding. Time-varying IPTW estimates propensity scores for each treatment decision and thereby allows treatment exposure, clinical characteristics, and concomitant medications to be updated throughout follow-up, including treatment changes and switching between glucose-lowering drug classes. Consequently, individuals could contribute follow-up under different treatment states during the study period. In contrast, standard propensity score matching or IPTW based on baseline treatment and covariates would primarily address differences in treatment assignment at baseline and would not account for subsequent changes in treatment and time-varying confounders. The longitudinal approach was therefore selected to align the analysis with the study question of treatment use during follow-up and the observed time-varying structure of the data.

The analysis was based on successive NDR registrations rather than predefined follow-up intervals. Because the exact timing of individual clinical treatment decisions was not directly observable, NDR registrations were used as analytical treatment assessment points. Treatment status at each registration was determined from reconstructed medication-use intervals based on dispensing data, as detailed in Supplementary Methods. Treatment probabilities were estimated at each observed registration using information available from the previous registration, and all time-varying predictors were therefore lagged by one registration to preserve the temporal ordering between predictors and treatment status. Because the timing of registrations varied between individuals, time-related variables were included in the treatment models to account for both follow-up duration and the interval between registrations.

Stabilized weights were calculated separately for SGLT2i, GLP-1RA, and DPP-4i. For each drug class, the numerator model included only treatment status at the previous registration (lagged exposure). For the first registration, lagged exposure was defined using dispensing information from the National Prescribed Drug Register (Supplementary Methods). The denominator model additionally included demographic characteristics and lagged information on treatment history, clinical risk factors, comorbidities, and concomitant medications, together with time-related variables including calendar year, time since baseline, and time since the previous registration. Calendar year was included to account for temporal changes in treatment assignment throughout the study period, reflecting evolving evidence, updates to clinical guidelines, and changing prescribing patterns in routine clinical practice. Continuous variables were modelled flexibly and clinically relevant interaction terms were included. Full model specifications are provided in the Supplementary Methods. Stabilized weights were truncated at the 0.5th and 99.5th percentiles.

The resulting weighted analyses estimate the association between treatment use during follow-up and stroke risk while accounting for measured time-varying confounding and treatment history. These estimates should not be interpreted as the effect of treatment initiation in drug-naïve individuals.

As a descriptive diagnostic, covariate balance before and after weighting was assessed using standardized mean differences (SMDs). Because treatment assignment and covariates were updated over time, balance was evaluated separately for the first six registrations for each drug class using Love plots.

The stabilized weights were applied in weighted time-varying Cox proportional hazards models to estimate hazard ratios for ischemic stroke, hemorrhagic stroke, and total stroke. Separate models were fitted for SGLT2 inhibitors, GLP-1 receptor agonists, and DPP-4 inhibitors using the corresponding drug-specific IPTW, with the drug class of interest included as a time-varying exposure. Robust standard errors clustered by individual were used to account for repeated registrations within individuals. The proportional hazards assumption was evaluated using Schoenfeld residuals, and no significant violations were detected.

Summary statistics of the IPTW weights were calculated separately within each imputed dataset and then averaged across imputations for descriptive presentation only. These pooled descriptive summaries of weights were not used in the analyses. For the outcome models, each imputed dataset was analysed separately using its own set of weights, and the resulting estimates and standard errors were then combined across imputations using Rubin's rules^30^ to obtain pooled effect estimates and confidence intervals.

In order to contextualize the findings, a systematic PubMed search was conducted from inception to June 23, 2026, using the terms (sodium-glucose cotransporter 2 OR SGLT2 OR SGLT-2 OR GLP1 OR GLP-1 OR glucagon-like peptide-1 OR DPP4 OR DPP-4 OR dipeptidyl peptidase-4) AND (stroke OR “cerebral infarction” OR “intracerebral hemorrhage”) AND diabetes, with the filter “Meta-Analysis” applied. The aim of this search was to identify published meta-analyses of randomised trials and observational studies, rather than to conduct a new systematic review or quantitative synthesis. Accordingly, no de novo meta-analysis was performed, and we did not assess publication bias, heterogeneity, risk of bias, or certainty of evidence using formal tools. The present comparisons should therefore be interpreted as contextual rather than as a formal evidence synthesis. Inclusion criteria were predefined as peer-reviewed meta-analyses of randomised trials or observational studies reporting stroke outcomes for SGLT2i, GLP-1RA, or DPP-4i. Meta-analyses without separate stroke estimates, published only as abstracts, or not peer-reviewed were excluded. Effect estimates with 95% confidence intervals from eligible studies were presented in forest plots, stratified by drug class and study design. Effect measures and definitions of stroke outcomes were retained as reported in the original meta-analyses and therefore varied across studies.

Ischemic stroke was the primary outcome and total stroke was a secondary outcome included in the original analysis plan. Hemorrhagic stroke was added as a post-hoc secondary outcome during peer review. No sensitivity analyses were performed. All statistical tests were two tailed, with alpha set at 5%. Statistical analyses were carried out using R version 4.4.2 (R Core Team [2025], R: A Language and Environment for Statistical Computing, R Foundation for Statistical Computing, Vienna, Austria).

### Role of the funding source

The funders had no involvement in study design, data collection, data analyses, data interpretation, or the writing of the report.

## Results

A total of 242,406 individuals with type 2 diabetes were registered for the first time in the NDR between January 1st, 2013 and December 31st, 2019. Of those 13,406 were excluded due to a prior stroke diagnosis, 57,079 due to insufficient data, and 4 because they had no observable follow-up time after interval construction. The final study population consisted of 171,917 individuals and a total of 1,617,462 clinical registrations (Fig. 1) with a maximum follow-up of 7 years [mean (SD) follow-up 3.0 (1.9) years]. Each patient contributed multiple follow-up visits with irregular and patient-specific intervals, as well as prescription refills occurring at varying times. In the final study population, the median (IQR) number of clinical visits per patient was 7.0 (4.0–12.0).

**Fig. 1:**
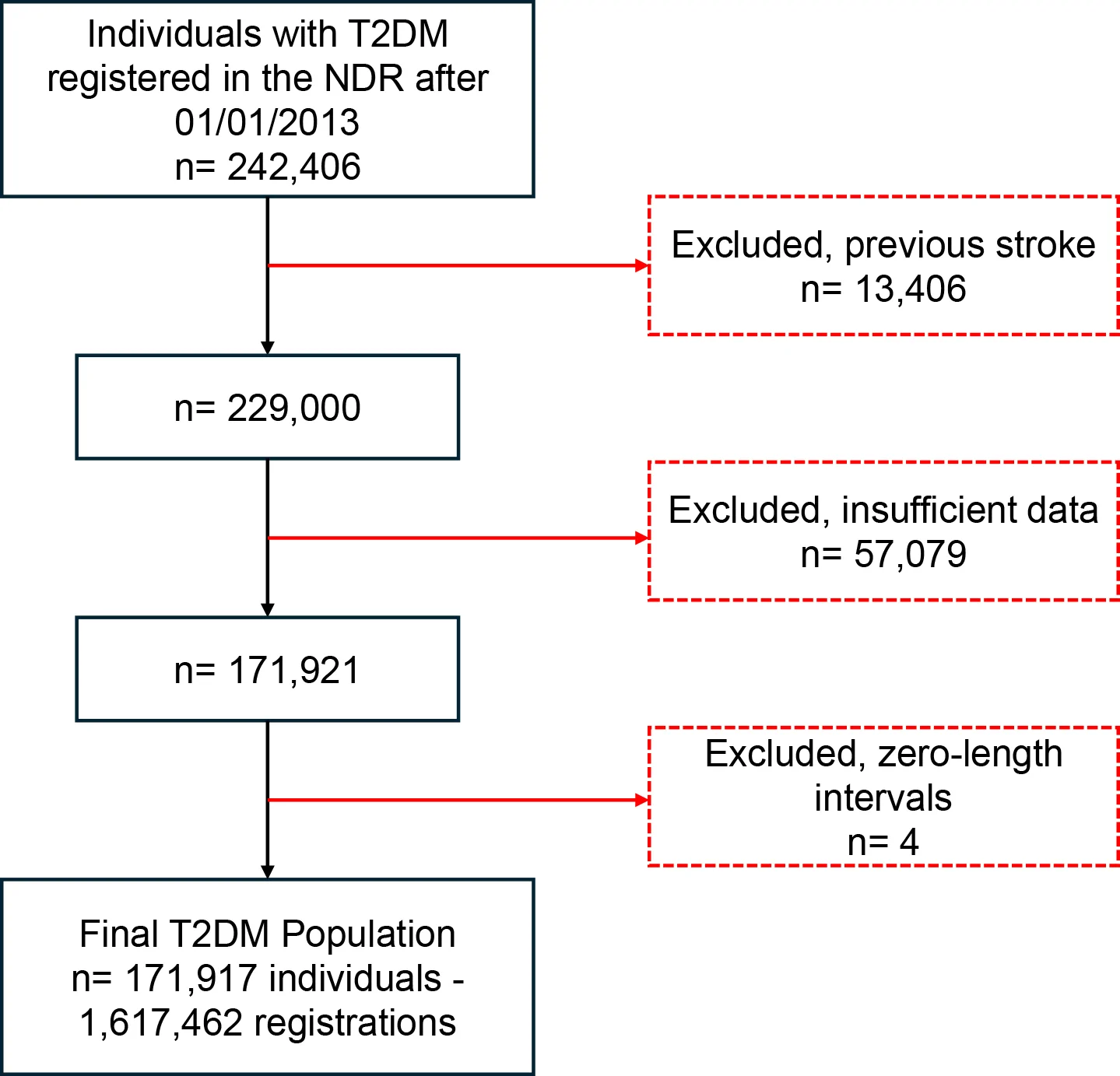
**Study profile.** T2DM, Type 2 Diabetes Mellitus; NDR, National Diabetes Register.

Baseline and longitudinal descriptive characteristics are presented in Table 1. At baseline, participants had a mean (SD) age of 62.7 (11.6), and 59.2% (101,837 of 171,917) were male. Overall, the most prevalent comorbidity was hypertension (33.7%, 57,860 of 171,917), followed by cancer (12.0%, 20,613 of 171,917) and atrial fibrillation (8.0%, 13,729 of 171,917). During the study period 7.9% (13,640 of 171,917) of individuals were at some point exposed to SGLT2i, 6.0% (10,287 of 171,917) to GLP-1RA and 13.2% (22,638 of 171,917) to DPP-4i. A total of 2734 individuals developed an ischemic stroke (1.59%; 5.29 per 1000 person-years) and 428 developed a hemorrhagic stroke (0.25%; 0.83 per 1000 person-years).

**Table 1:** Descriptive Characteristics of the (pre-imputation) study population.

	Descriptive summary	
	Baseline N = 171,917	Longitudinal N = 171,917
Number of registrations, median (IQR)	NA (NA, NA)	7.0 (4.0, 12.0)
Follow-up time, years	NA (NA)	3.0 (1.9)
Age, years	62.7 (11.6)	64.2 (11.6)
Sex		
Male	101,837 (59.2%)	101,837 (59.2%)
Female	70,080 (40.8%)	70,080 (40.8%)
HbA1c, mmol/mol	56.3 (19.7)	51.0 (11.1)
Systolic blood pressure, mmHg	136.2 (16.5)	134.6 (12.1)
Body mass index, kg/m^2^	31.0 (5.8)	30.4 (5.6)
LDL cholesterol, mmol/L	3.1 (1.1)	2.9 (0.9)
eGFR, mL/min/1.73 m^2^	86.9 (23.8)	85.7 (22.4)
Albuminuria		
No	74,417 (84.8%)	115,396 (72.8%)
Microalbuminuria	10,781 (12.3%)	34,809 (22.0%)
Macroalbuminuria	2576 (2.9%)	8250 (5.2%)
Smoking	22,083 (16.3%)	33,852 (19.7%)
Physical activity		
Never	16,943 (14.0%)	27,308 (15.9%)
<1/week	17,346 (14.3%)	22,385 (13.0%)
1–2/week	23,329 (19.3%)	32,063 (18.7%)
3–5/week	26,253 (21.7%)	37,647 (21.9%)
Daily	37,167 (30.7%)	52,505 (30.5%)
Education		
Lower	51,756 (30.1%)	51,381 (29.9%)
Middle	80,142 (46.6%)	80,230 (46.7%)
Higher	40,019 (23.3%)	40,306 (23.4%)
Income		
Lower	54,342 (31.6%)	43,405 (25.2%)
Middle	57,788 (33.6%)	53,201 (30.9%)
Higher	59,787 (34.8%)	75,311 (43.8%)
Cancer	20,613 (12.0%)	28,329 (16.5%)
Dementia	529 (0.3%)	1540 (0.9%)
Alcohol/drug abuse	9850 (5.7%)	11,943 (6.9%)
Hypertension	57,860 (33.7%)	75,348 (43.8%)
Atrial fibrillation	13,729 (8.0%)	18,457 (10.7%)
Ischemic heart disease	12,953 (7.5%)	15,568 (9.1%)
Heart failure	8927 (5.2%)	12,792 (7.4%)
Respiratory conditions	13,474 (7.8%)	16,949 (9.9%)
Vascular conditions	4604 (2.7%)	6565 (3.8%)
Insulin	11,185 (6.5%)	23,353 (13.6%)
SGLT2 inhibitors	1005 (0.6%)	13,640 (7.9%)
GLP-1 receptor agonists	994 (0.6%)	10,287 (6.0%)
DPP-4 inhibitors	4069 (2.4%)	22,638 (13.2%)
Other glucose-lowering medications	82,001 (47.7%)	132,625 (77.1%)
Anticoagulants	12,433 (7.2%)	22,269 (13.0%)
Antiplatelets	32,343 (18.8%)	42,351 (24.6%)
Antihypertensives	95,144 (55.3%)	120,637 (70.2%)
Statins	64,209 (37.3%)	110,487 (64.3%)
Diuretics	30,025 (17.5%)	44,405 (25.8%)
Beta blockers/Antiarrhythmics/Glycosides	51,095 (29.7%)	69,188 (40.2%)

Continuous variables are presented as mean (SD), except number of registrations, which is presented as median (IQR). Categorical variables are presented as n (%). Longitudinal continuous summaries are means of individual-level means; binary variables represent ever during follow-up; physical activity represents the most frequent category; education, income, and albuminuria use the maximum recorded category. Missing data across registrations: HbA1c: 451824 (27.9%); Systolic blood pressure: 444858 (27.5%); Body mass index: 683181 (42.2%); LDL cholesterol: 833302 (51.5%); eGFR: 696070 (43%); Smoking: 661351 (40.9%); Physical activity: 818362 (50.6%); Albuminuria: 910484 (56.3%). Abbreviations: SD, standard deviation; IQR, interquartile range; HbA1c, glycated haemoglobin A1c; LDL, low-density lipoprotein; eGFR, estimated glomerular filtration rate; SGLT2, sodium-glucose cotransporter 2; GLP-1, glucagon-like peptide-1; DPP-4, dipeptidyl peptidase-4; NA, not applicable.

Averaged descriptive summaries of the stabilized weights across imputations are presented in Table 2. Weight distributions were centered close to 1 for all three drug classes, with mean (SD) weights of 0.97 (0.26) for SGLT2i, 0.99 (0.25) for GLP-1RA, and 1.02 (0.47) for DPP-4i. Histograms of the stabilized weights are presented in Supplementary Figure S3. Weighting generally reduced standardized mean differences across the first six registrations for all three drug classes (Supplementary Figures S4–S6).

**Table 2:** Weight distribution and weighted hazard ratios.

Exposure	Weight distribution		HR (95% CI)		
	Mean (SD)	Range	Ischemic stroke	Hemorrhagic stroke	Total stroke
**SGLT2i**	0.97 (0.26)	0.14–3.50	0.94 (0.65–1.37)	0.51 (0.14–1.83)	0.90 (0.63–1.28)
**GLP-1RA**	0.99 (0.25)	0.14–3.44	0.46 (0.29–0.72)	0.80 (0.28–2.28)	0.51 (0.33–0.79)
**DPP-4i**	1.02 (0.47)	0.18–5.93	1.17 (0.95–1.42)	1.62 (0.97–2.72)	1.20 (0.99–1.45)

HR, hazard ratio; CI, confidence interval; SGLT2i, sodium-glucose cotransporter-2 inhibitor; GLP-1RA, glucagon-like peptide-1 receptor agonist; DPP-4i, dipeptidyl peptidase-4 inhibitor; SD, standard deviation. Reference category: non-use of the drug class of interest.

Results from the weighted Cox regression analyses are presented in Table 2. GLP-1 RA use was associated with a lower hazard of ischemic stroke (HR 0.46, 95% CI 0.29–0.72) and total stroke (HR 0.51, 95% CI 0.33–0.79). No statistically significant association was observed for hemorrhagic stroke (HR 0.80, 95% CI 0.28–2.28). SGLT2i use was not associated with ischemic stroke (HR 0.94, 95% CI 0.65–1.37), hemorrhagic stroke (HR 0.51, 95% CI 0.14–1.83), or total stroke (HR 0.90, 95% CI 0.63–1.28). DPP-4i use was not associated with ischemic stroke (HR 1.17, 95% CI 0.95–1.42), hemorrhagic stroke (HR 1.62, 95% CI 0.97–2.72), or total stroke (HR 1.20, 95% CI 0.99–1.45). Across all three medication classes, estimates for hemorrhagic stroke were imprecise, reflecting the relatively small number of events.

The PubMed search returned 274 articles, of which titles and abstracts were screened. This resulted in 102 full texts being assessed, and 91 meta-analyses of RCTs^7^, ^8^, ^9^^,^^12^^,^^31^, ^32^, ^33^, ^34^, ^35^, ^36^, ^37^, ^38^, ^39^, ^40^, ^41^, ^42^, ^43^, ^44^, ^45^, ^46^, ^47^, ^48^, ^49^, ^50^, ^51^, ^52^, ^53^, ^54^, ^55^, ^56^, ^57^, ^58^, ^59^, ^60^, ^61^, ^62^, ^63^, ^64^, ^65^, ^66^, ^67^, ^68^, ^69^, ^70^^,^^71^, ^72^, ^73^, ^74^, ^75^, ^76^, ^77^, ^78^, ^79^, ^80^, ^81^, ^82^, ^83^, ^84^, ^85^, ^86^, ^87^, ^88^, ^89^, ^90^, ^91^, ^92^, ^93^, ^94^, ^95^, ^96^, ^97^, ^98^, ^99^, ^100^, ^101^, ^102^, ^103^, ^104^, ^105^, ^106^, ^107^, ^108^, ^109^, ^110^, ^111^, ^112^, ^113^ and observational studies^12^^,^^14^^,^^15^^,^^107^^,^^114^^,^^115^ being included. Meta-analyses of RCTs for SGLT2i consistently reported no effect on stroke risk, with effect sizes ranging from 0.84 to 1.30, whereas observational meta-analyses suggested a protective effect, with estimates between 0.75 and 0.87. For GLP-1RA both RCTs and observational meta-analyses consistently showed a protective effect, with estimates ranging from 0.80 to 0.91. For DPP-4i RCTs indicated a neutral effect (0.77–1.03), and only one meta-analysis including combined observational and trial data was identified. The effect sizes with 95% CIs of the included meta-analyses, along with the findings of the present study, are presented in Fig. 2.

**Fig. 2:**
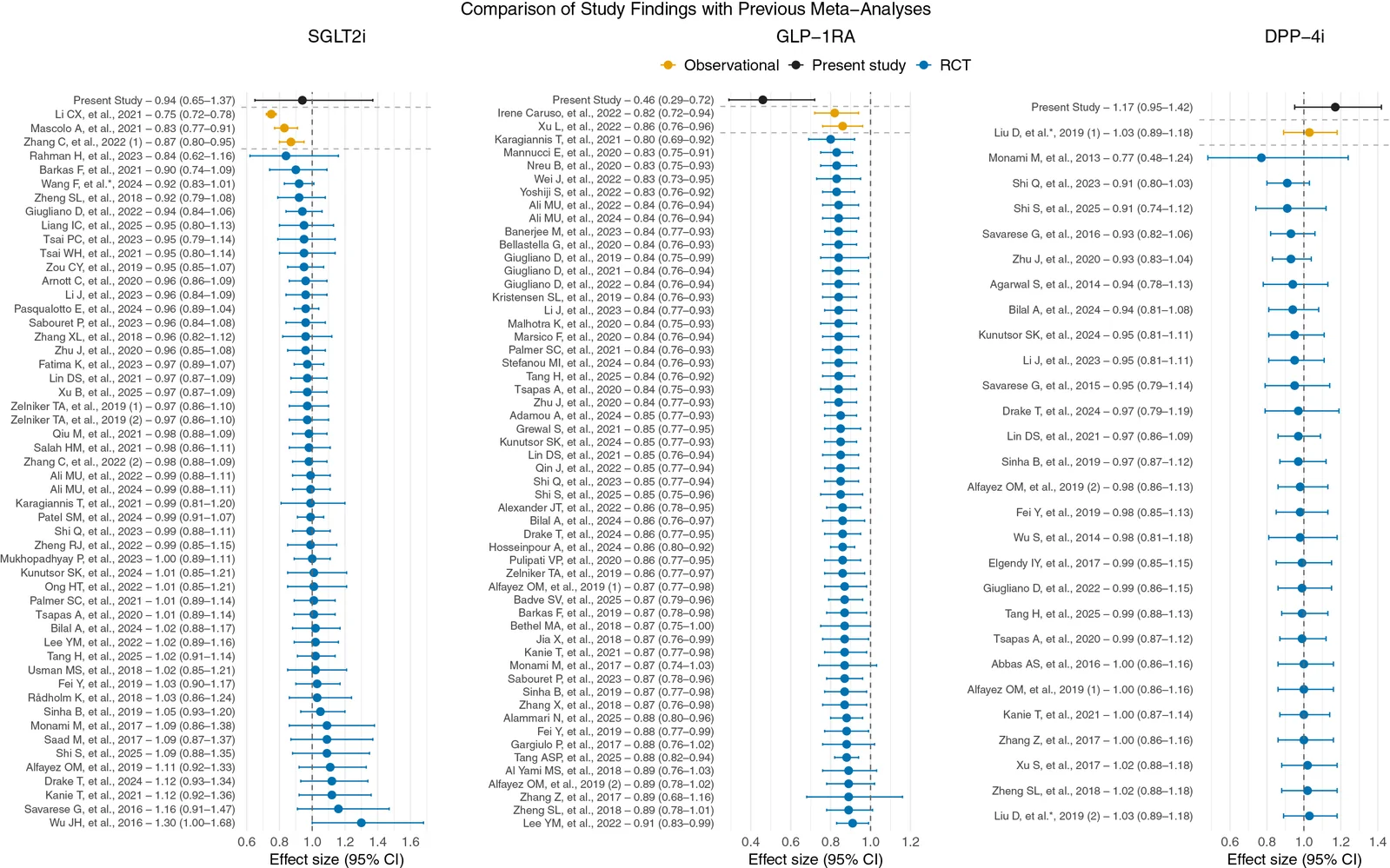
**Present study estimates relative to meta-analyses of randomized controlled trials and observational studies.** Effect estimates and 95% confidence intervals for stroke outcomes associated with SGLT2 inhibitors (SGLT2i), glucagon-like peptide-1 receptor agonists (GLP-1RA), and dipeptidyl peptidase-4 inhibitors (DPP-4i) in previously published meta-analyses, together with the ischemic stroke hazard ratios from the present study. Stroke definitions and effect measures varied across meta-analyses. Estimates are stratified by study design as randomised controlled trial (RCT) meta-analyses and observational meta-analyses. The comparison is intended for contextualisation; estimates across studies may represent different treatment contrasts and are not directly quantitatively comparable. ∗ Included both observational and RCT data.

## Discussion

This study aimed to examine stroke risk associated with SGLT2i, GLP-1RA, and DPP-4i use in individuals with type 2 diabetes, using nationwide longitudinal data. In a cohort of 171,917 individuals with type 2 diabetes, GLP-1RA were associated with lower risk of both ischemic and total stroke, while SGLT2i and DPP-4i showed no clear associations with stroke risk. These findings are directionally consistent with results reported in RCTs, providing complementary real-world evidence on the cerebrovascular benefits of GLP-1RA and the neutral effects of SGLT2i and DPP-4i.

Several placebo-controlled RCTs have examined the effect of GLP-1RA on stroke risk and consistently demonstrated a 10–20% reduction.^7^, ^8^, ^9^^,^^73^^,^^79^ A meta-analysis of real-world studies reported similar benefits.^114^ However individual observational analyses have been inconsistent, with estimates ranging from a 70% reduction in stroke hazard,^116^ to non-significant findings.^117^^,^^118^ In the present study, GLP-1RA use was associated with lower hazards of ischemic stroke. While the magnitude of the observed association was greater than that reported in most RCTs, the direction of effect was consistent with the overall evidence base. For SGLT2i, placebo-controlled RCTs have generally reported neutral effects on stroke,^7^, ^8^, ^9^^,^^53^ while meta-analyses of observational studies often suggest a protective effect, particularly in comparisons with other glucose-lowering agents.^12^^,^^14^^,^^15^ Other real-world studies comparing SGLT2i directly with GLP-1RA have typically found no significant differences.^10^^,^^11^ In our analysis, SGLT2i showed neutral effects, in line with the RCT evidence. DPP-4i have likewise been consistently neutral in RCTs,^7^, ^8^, ^9^ although some observational studies have suggested protective effects.^16^^,^^17^ Real-world evidence was included in only one^107^ of the identified meta-analyses, which also incorporated RCTs and reported a neutral effect. In the present study, DPP-4i showed no clear association with ischemic stroke, consistent with trial evidence.

The agreement between our SGLT2i findings and randomized trial evidence, despite contrasting with several observational meta-analyses, may partly reflect differences in methodology. The majority of prior real-world studies have relied on baseline-only adjustment, which is suitable for an analysis of initial treatment akin to intention-to-treat analysis. However, adjustment for baseline variables may not adequately capture confounding of dynamic treatment regimes that evolve during follow-up. When exposures and covariates change over time, such static approaches can yield biased estimates.^29^^,^^119^ As treatment switching is common in clinical practice, an intention-to-treat estimator has limited information for long-term follow-up and does not directly address the question “what is the effect if individuals actually fill prescriptions?” Differences in length of follow-up, type of estimand, treatment adherence and persistence, study populations, outcome ascertainment, and residual confounding may all contribute to differences between observational studies and randomized trials. By incorporating longitudinal data with repeated measures, the present study explicitly considers treatment changes and evolving clinical characteristics that may influence observational estimates.

The stronger association observed for GLP-1RA compared with most randomized trials may have several explanations. Most trials report intention-to-treat analyses, which estimate the effect of treatment assignment regardless of subsequent adherence or treatment changes. In contrast, the present analysis incorporated treatment use throughout follow-up, including treatment discontinuation and switching. As a result, the estimated associations are not directly comparable and may differ when adherence changes over time. Differences in study populations, treatment patterns, residual confounding, and effect modification may also contribute. Consequently, the larger effect estimates observed in the present study should not be attributed to any single explanation and should not be interpreted as evidence that GLP-1RA are more effective in routine clinical practice than suggested by randomized trials.

Several biological mechanisms may explain the observed reduction in ischemic stroke risk associated with GLP-1 receptor agonists.^120^, ^121^, ^122^ Beyond glucose lowering, GLP-1RA exert multiple cardiometabolic effects that influence established cerebrovascular risk pathways, including reductions in systolic blood pressure, body weight, and post-prandial lipaemia, as well as improvements in endothelial function and vascular inflammation.^120^ Experimental and clinical studies also suggest anti-atherosclerotic effects mediated through reduced oxidative stress, improved nitric oxide bioavailability, and modulation of macrophage activity within atherosclerotic plaques, which may contribute to stabilization of vulnerable vascular lesions.^120^ In addition to systemic vascular effects, GLP-1 receptors are expressed in the central nervous system, and preclinical models indicate potential direct neuroprotective properties, including attenuation of excitotoxicity, reduced neuroinflammation, decreased apoptotic signalling, and promotion of angiogenesis.^120^^,^^122^ In contrast, DPP-4 inhibitors increase endogenous incretin levels only modestly and do not appear to produce the same magnitude of metabolic or vascular effects as GLP-1RA.^123^ Similarly, SGLT2 inhibitors produce substantial cardiovascular and renal benefits through mechanisms including osmotic diuresis, natriuresis, reductions in blood pressure and arterial stiffness, and improved cardiac loading conditions^124^^,^^125^; however, these effects may not directly target atherosclerotic or thromboembolic pathways most relevant to ischemic stroke. In addition, SGLT2 inhibition is associated with increases in hematocrit and hemoconcentration,^125^ which have been hypothesized to offset potential cerebrovascular benefits. Together, these mechanistic differences provide a biological explanation for the divergence in stroke outcomes between GLP-1RA and the other glucose-lowering classes examined.

Evidence regarding hemorrhagic stroke is considerably more limited than for ischemic stroke. For GLP-1RA meta-analyses of RCTs have reported no significant association with hemorrhagic stroke,^87^^,^^90^^,^^93^^,^^126^ and a similar neutral pattern has been reported for DPP-4i.^126^ These findings are consistent with the present study, in which neither GLP-1RA nor DPP-4i showed a clear association with hemorrhagic stroke. For SGLT2i, some meta-analyses have suggested a reduction in hemorrhagic stroke risk.^37^^,^^70^ In the present study, no clear association was observed. Although the point estimate was 0.5 the confidence interval was wide, reflecting the limited number of hemorrhagic stroke events, and the findings neither clearly support nor exclude a protective effect.

This study has several important strengths. It was based on a large, nationally representative cohort with long follow-up and linkage across high-quality Swedish registers, minimizing selection bias. The longitudinal design allowed repeated updating of laboratory values, comorbidities, and medication use, making it possible to address time-varying confounding and account for treatment switching. The estimated associations reflect treatment use during follow-up and address the question “what is the average hazard ratio of treatment at any time-point?” Estimates are qualitatively comparable with intention-to-treat estimates reported in most randomized trials but are not directly comparable quantitatively.

This longitudinal approach was chosen because it aligned with the research question and the time-varying structure of the data. A baseline IPTW analysis would instead have estimated associations according to initial treatment status, without updating treatment classification for subsequent initiation, discontinuation, or switching, and might therefore have yielded different estimates as actual treatment patterns diverged during follow-up. However, the longitudinal approach introduces additional assumptions and modelling complexity, including correct model specification and no unmeasured confounding, which may themselves introduce bias if not adequately satisfied. Because we did not perform a parallel baseline analysis, we cannot determine the magnitude or direction of differences between the approaches or conclude empirically that the present approach produced less biased estimates. No upper age limit was imposed to reflect routine clinical practice and ensure generalisability across all ages. Even though age was adjusted for analytically, some residual age-related confounding cannot be excluded. The high degree of missingness in several clinically important variables represents an important limitation, as a substantial proportion of the information used to control for confounding was derived from the imputation models. Consequently, the estimates are more dependent on the adequacy of the imputation models and assumptions regarding the missingness mechanism. If missingness remained related to unobserved clinical characteristics associated with treatment selection and stroke risk, the imputation models may not have adequately represented the unobserved values, potentially resulting in residual confounding and biased effect estimates. The magnitude and direction of such bias cannot be determined. Although a larger number of imputations may have further reduced error, five imputations were used to balance statistical efficiency with the substantial computational demands of two-level multiple imputation with random effects in this large longitudinal dataset. The epidemiological definition of type 2 diabetes used in this study has not been formally validated and may therefore have introduced some misclassification. More specifically, the inclusion of some individuals with late onset type 1 diabetes diagnosed after the age of 40 cannot be excluded. However, this definition has been widely applied in large NDR-based epidemiological studies^1^ and helps mitigate the potential impact of incorrect or inconsistent registry coding. Additionally, given the inclusion of individuals that were first registered in the NDR from 2013 onwards, the findings may be less generalizable to individuals with longer diabetes duration. An alternative new-user design could have included individuals already registered in the NDR and defined cohort entry according to initiation of each treatment of interest. Such a design would have included a different study population and estimated associations with treatment initiation rather than treatment use throughout follow-up. Consequently, it might have produced different effect estimates, although the magnitude and direction of such differences cannot be determined from the present analysis. Drug exposure was defined based on dispensed prescriptions, which may not fully reflect adherence, and medication intervals were reconstructed using a predefined procedure that may not capture actual drug utilization. Furthermore, the exact timing of clinical treatment decisions relative to clinical measurements was not available; although lagging clinical predictors by one registration ensured that the information used in the treatment models preceded the treatment status being modelled, this represents an analytical approximation of the underlying clinical treatment process. Although treatment history and concurrent therapies were accounted for analytically in IPTW, the MSM assumed that only concurrent treatment affects the hazard. Residual biological carry-over effects from prior treatments cannot be completely excluded. The relatively small number of hemorrhagic stroke events limited the precision of hemorrhagic stroke analyses. The analyses used cause-specific Cox models, with death and competing stroke subtypes treated as censoring events; consequently, the estimated hazard ratios should be interpreted as cause-specific associations. Finally, the comparison with previously published meta-analyses was intended to contextualise the findings rather than to constitute a comprehensive systematic review. We did not perform duplicate screening, formal quality assessment, or de novo meta-analysis of individual studies, and the literature search was restricted to identifying published meta-analyses. As such, this component should be interpreted as a qualitative comparison within the existing evidence base and not as a formal systematic review. Overall, as with all observational studies the present analysis may still be affected by sources of bias such as residual confounding, exposure misclassification, and model misspecification, and the findings should therefore be interpreted with appropriate caution. Accordingly, although differences in estimands, treatment adherence and persistence, and study populations may partly explain the stronger GLP-1RA association relative to most randomized trials, the possibility that residual confounding or other sources of bias inherent to observational studies also contributed to the larger observed effect estimates cannot be excluded. As this study is based on nationwide data from Sweden the findings are most likely generalisable to settings with similar universal health-care systems and prescribing practices.

In this nationwide longitudinal study, GLP-1RA were associated with a reduced risk of stroke, while SGLT2i and DPP-4i showed no clear effects. These findings are consistent with results from RCTs and provide complementary evidence from routine clinical care. Overall, GLP-1RA may be considered as part of strategies to reduce cerebrovascular risk in individuals with type 2 diabetes. At the same time, the established benefits of SGLT2i and DPP-4i for other cardiovascular and renal outcomes should remain central in clinical decisions. It may be most appropriate for treatment choices to be informed by the totality of available evidence, recognizing that the benefits and limitations of each drug class should be thoughtfully weighed in decisions affecting both individuals and public health.

## Contributors

All authors had final responsibility for the decision to submit for publication. AM, TA, AV, and KSS accessed and verified the underlying data. **AM:** conceptualization, methodology, software, analysis, data curation, writing—original draft, visualization, project administration; **TA:** conceptualization, methodology, writing—review & editing, supervision, funding acquisition; **DB:** conceptualization, methodology, writing—review & editing, supervision; **KS:** methodology, writing—review & editing; **BE:** writing—review & editing; **MvE:** writing—review & editing; **AV:** conceptualization, methodology, data curation, writing—review & editing, supervision, funding acquisition; **KSS:** conceptualization, writing—review & editing, supervision, funding acquisition.

## Data sharing statement

According to the Swedish regulations, data can only be used in accordance with the application for this study that was approved by the Swedish Ethical Review Authority. Requests to access the dataset can be submitted by qualified researchers to the authors (contact Professor Katharina S. Sunnerhagen, email: ks.sunnerhagen@neuro.gu.se).

## Declaration of interests

BE reports personal fees from Novo Nordisk, Eli Lilly, Sanofi, Abbott, and Amgen. All other authors declare no competing interests.
