# Supplementary material for: The effect of SGLT2 inhibitors, GLP-1 receptor agonists, or DPP-4 inhibitors on stroke risk in type 2 diabetes: a nationwide longitudinal cohort study in Sweden with contextualisation analysis
Source: eClinicalMedicine. 2026 Sep 23;100:104220. doi: 10.1016/j.eclinm.2026.104220 (PMC13634272; doi:10.1016/j.eclinm.2026.104220)

ONLINE SUPPLEMENT

The effect of SGLT2 inhibitors, GLP-1 receptor agonists, or DPP-4 inhibitors on stroke risk in Type 2 Diabetes: a nationwide longitudinal cohort study in Sweden with contextualisation analysis

Anastasios Mavridis[1], MD; Tamar Abzhandadze[1,2] PhD; Dongni Buvarp[1,3,4] MD, PhD; Koen Simons[5] PhD; Björn Eliasson[4,6] MD, PhD; Mia von Euler[7] MD, PhD; Adam Viktorisson[1,4] MD, PhD; Katharina S Sunnerhagen[1,4] MD, PhD

Affiliations

1. Department of Clinical Neuroscience, Institute of Neuroscience and Physiology, Sahlgrenska Academy, University of Gothenburg, Sweden

2. Department of Occupational Therapy and Physiotherapy, Sahlgrenska University Hospital, Gothenburg, Sweden

3. Department of Neurology, Sahlgrenska University Hospital, Gothenburg, Sweden

4. The Sahlgrenska University Hospital, Gothenburg, Sweden

5. Biostatistics, School of Public Health and Community Medicine, Institute of Medicine, Sahlgrenska Academy, University of Gothenburg, Gothenburg, Sweden

6. The National Diabetes Register, Center of Registers, Gothenburg, Sweden

7. Sophiahemmet University, Stockholm, Sweden

Index

Supplementary Methods 3
- Definition of active medication intervals 3
- Definition of lagged exposure 3
- IPTW model specification 4

Supplementary Figures 6
- Supplementary Figure 1. Convergence Trace Plots for 2-Level Predictive Mean Matching Imputations........ 6
- Supplementary Figure 2. Density plots of original and imputed distributions of imputed variables........ 7
- Supplementary Figure 3. Distribution of Stabilized Weights (Stacked Across Imputations) 8
- Supplementary Figure 4. Standardized mean differences before and after inverse probability of treatment weighting for SGLT2 inhibitor use across the first six NDR registrations. 9
- Supplementary Figure 5. Standardized mean differences before and after inverse probability of treatment weighting for GLP-1 receptor agonist use across the first six NDR registrations. 10
- Supplementary Figure 6. Standardized mean differences before and after inverse probability of treatment weighting for DPP-4 inhibitor use across the first six NDR registrations. 11

Supplementary Methods

Definition of active medication intervals

Because the Prescribed Drug Register records dispensing dates rather than actual treatment periods, medication use intervals were estimated from dispensing information. These intervals were used to define time-varying treatment exposure throughout follow-up and to identify treatment status at each NDR registration.

Data on dispensed prescriptions was obtained from the Swedish National Prescribed Drug Register. For each record, the available information included the date of collection, the number of packages dispensed, and the expected duration (in days) of each package, based on its Defined Daily Dose (DDD). The expected duration of each prescription was calculated by multiplying the number of packages by the expected days per package, yielding an estimated end date for each prescription.

Medication use intervals were defined using a three-step procedure:

1) Overlap merging: If the estimated end date of one prescription overlapped with the start date of the next, the two intervals were merged into a single continuous interval.
2) Pattern-based merging: Because exact individual dosing was unknown and consistent refill patterns were observed for certain drugs (e.g., regular intervals between prescriptions without direct overlap), we calculated, for each drug, the median and interquartile range (IQR) of the time gaps between consecutive prescriptions. A merging threshold was set at median + IQR. If the gap between two prescriptions for the same drug was within this threshold, the intervals were merged.
3) Grace period: A final 30-day grace period was applied to account for minor deviations in prescription refills.

This approach ensured that continuous treatment episodes were captured while allowing for variability in dispensing patterns and patient adherence. Active treatment status for each drug class was determined at every NDR registration based on whether the registration date fell within an active medication interval.

Definition of lagged exposure

Lagged exposure for each medication of interest was defined as the exposure status at the previous registration. By definition, this resulted in missing values for the first registration of each individual. These were handled using the following rules:

1) No prior dispensing record: If the individual had no record in the National Prescribed Drug Register before baseline, lagged exposure variables were set to 0 (no prior use).
2) Recent initiation before baseline: If any medication interval started ≤90 days before baseline, it was assumed to be part of the same treatment episode as the baseline registration, and lagged exposure was set to 0.

3) Earlier use before baseline: If any medication interval started >90 days before baseline, lagged exposure was set to 1, reflecting prior use.

A 90-day threshold was chosen because it aligns with common three-month prescription refill patterns in Sweden.

IPTW model specification

IPTW within a marginal structural model (MSM) framework was used to account for time-varying confounding. In the time-invariant setting IPTW emulates one-off randomisation to immutable groups such as initial treatment or intention to treat. An ideal randomised controlled trial to study time-varying treatments can be imagined as repeatedly randomising treatment at each follow-up, thus ensuring treatment is not confounded by concurrent covariates, nor by history of covariates. A statistical model for such data requires a structural assumption, for instance a model for the hazard of an event may include the concurrent and previous treatment and an interaction between them. A MSM including only concurrent treatment imposes a stronger structural assumption.

The IPTW analysis was based on successive NDR registrations rather than predefined follow-up intervals. Each registration represented a potential treatment decision point at which the probability of the observed treatment status was estimated. Treatment exposure and covariate information were therefore updated whenever a new registration became available. Because the timing of registrations varied between individuals, time since the previous registration was incorporated into the treatment models.

To better reflect the temporal ordering between predictors and treatment assignment, treatment probabilities were estimated using information available before the treatment status being modelled. Accordingly, all time-varying predictors included in the treatment models were lagged by one registration.

Treatment status at each registration was modelled using logistic regression. Separate stabilized weight models were estimated for SGLT2i, GLP-1RA, and DPP-4i. For each drug class, the numerator model included only lagged exposure to the drug under study.

The same denominator model specification was used for all three drug classes and included:

- Sex
- Educational level
- Age at baseline
- Lagged SGLT2i use
- Lagged GLP-1RA use
- Lagged DPP-4i use
- Lagged insulin use
- Lagged HbA1c
- Lagged systolic blood pressure
- Lagged BMI
- Lagged LDL cholesterol
- Lagged eGFR
- Lagged hypertension
- Lagged ischemic heart disease
- Lagged heart failure
- Lagged atrial fibrillation

- Lagged vascular disease
- Lagged anticoagulant use
- Lagged antiplatelet use
- Lagged antihypertensive use
- Lagged statin use
- Calendar year
- Time since baseline
- Time since the previous registration

Age at baseline, HbA1c, systolic blood pressure, BMI, LDL cholesterol, eGFR, and time since baseline were modelled using natural splines with three degrees of freedom.

The following interaction terms were included:

- HbA1c × SGLT2i
- HbA1c × GLP-1RA
- HbA1c × DPP-4i
- HbA1c × insulin
- BMI × GLP-1RA
- eGFR × SGLT2i
- Calendar year × SGLT2i
- Calendar year × GLP-1RA
- Calendar year × DPP-4i

Exposure indicators were not mutually exclusive. Individuals could be simultaneously classified as exposed to multiple glucose-lowering drug classes if active medication intervals overlapped. Stabilized weights were truncated at the 0.5th and 99.5th percentiles.

The stabilized weights were applied in weighted time-varying Cox proportional hazards models to estimate hazard ratios for ischemic stroke, hemorrhagic stroke, and total stroke. In addition to the proportional hazards assumption, a structural assumption is imposed: only concurrent treatment affects the hazard.

Supplementary Figures

Supplementary Figure 1. Convergence Trace Plots for 2-Level Predictive Mean Matching Imputations

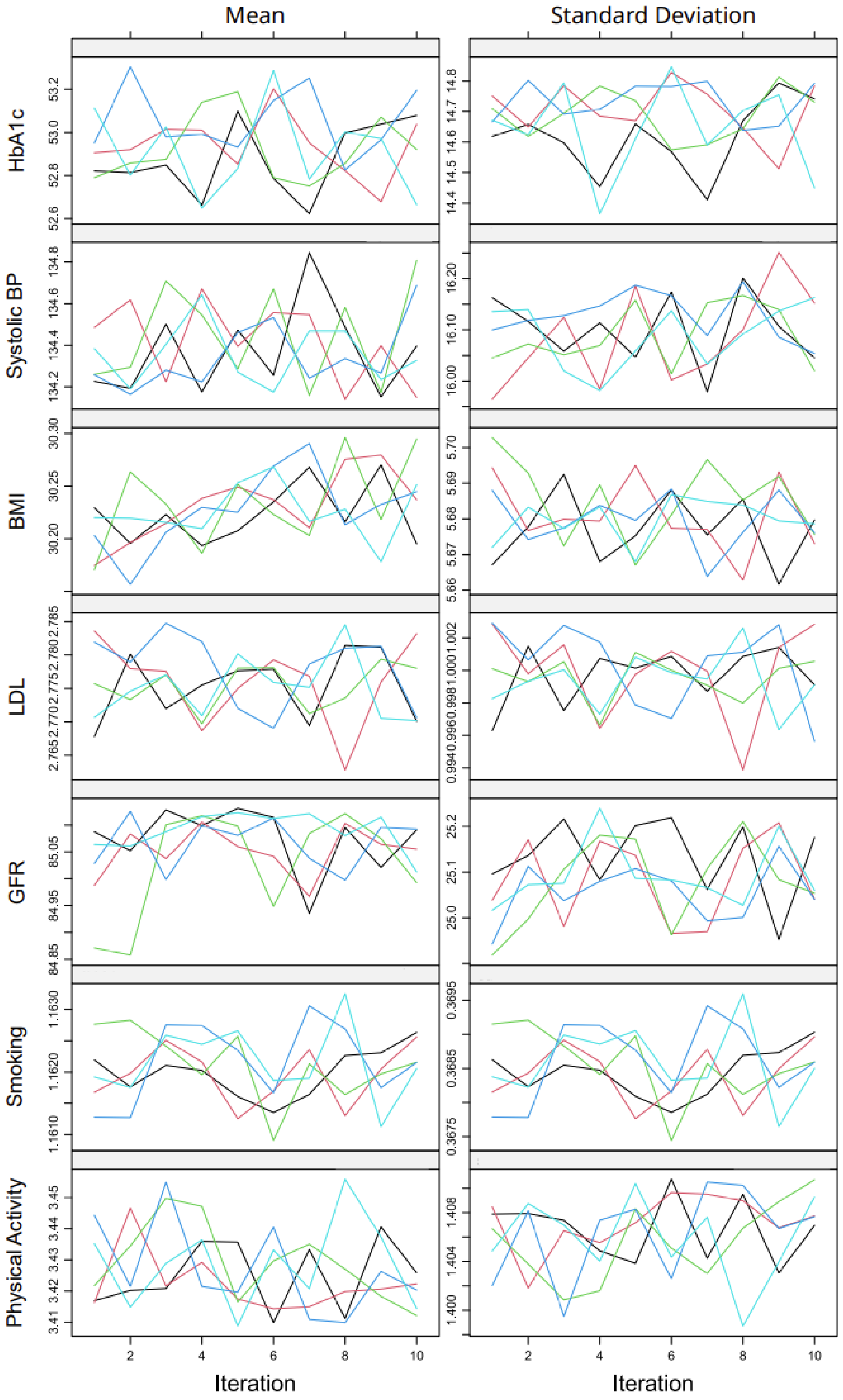

Supplementary Figure 2. Density plots of original and imputed distributions of imputed variables

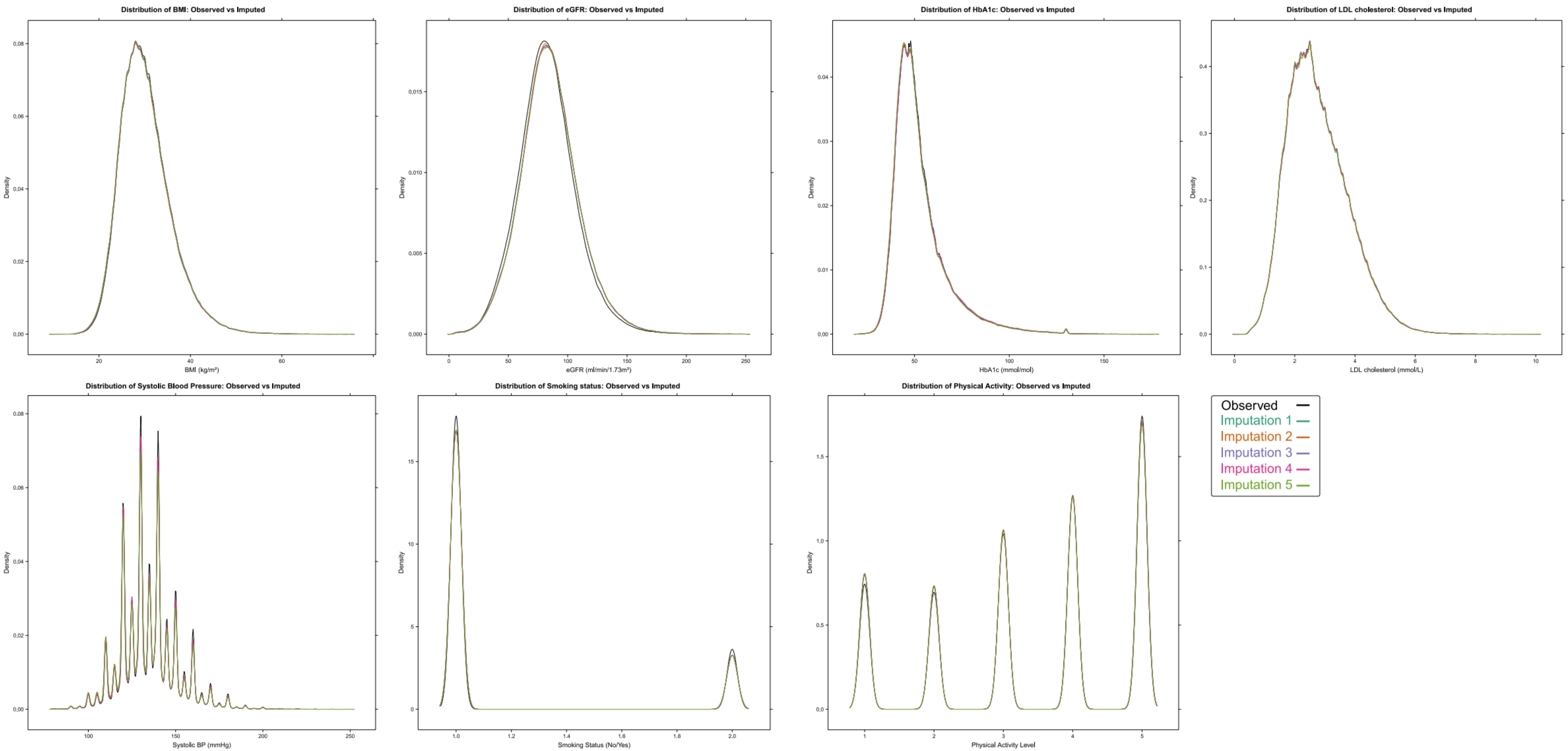

Supplementary Figure 3. Distribution of Stabilized Weights (Stacked Across Imputations)

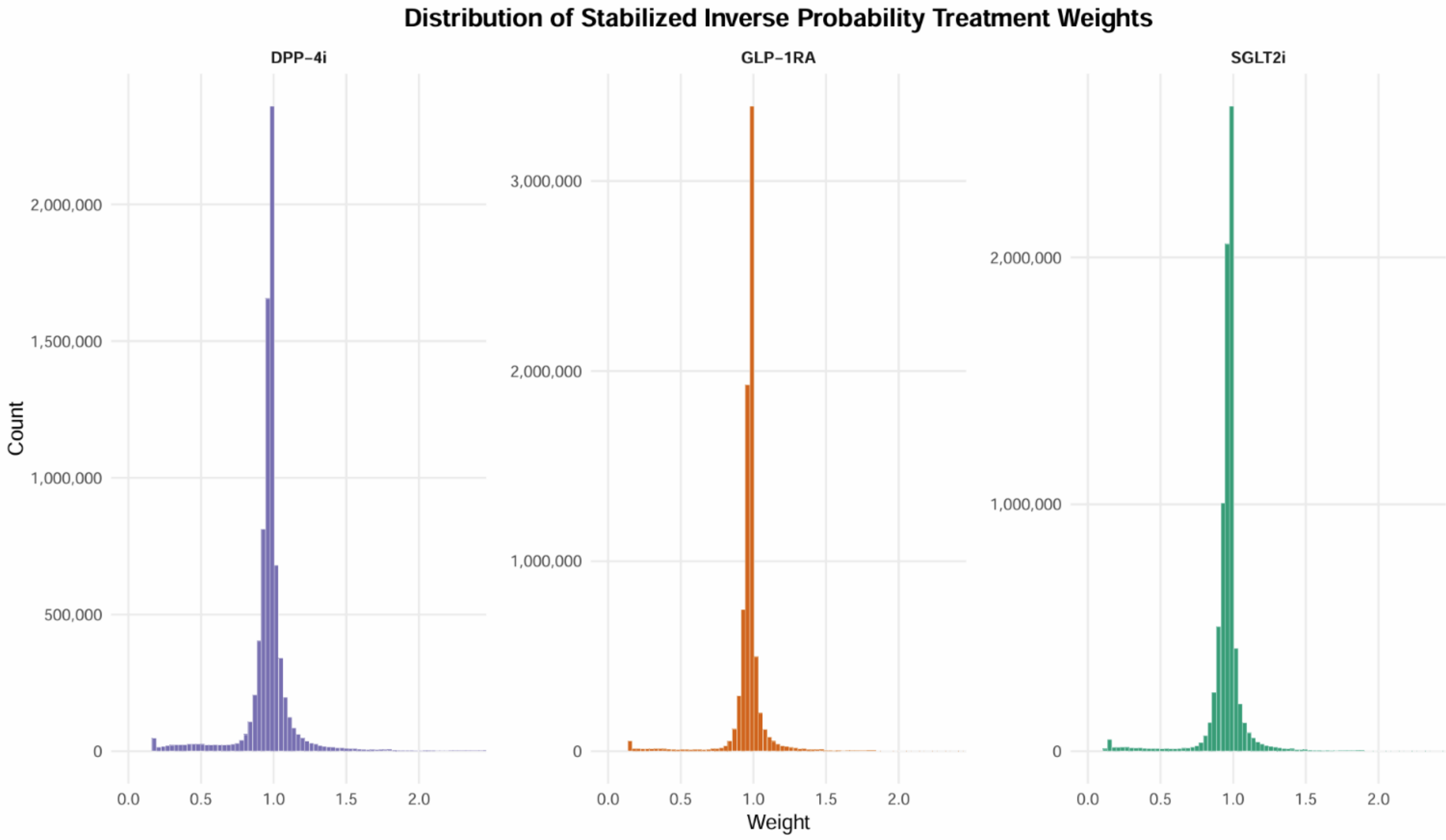

Supplementary Figure 4. Standardized mean differences before and after inverse probability of treatment weighting for SGLT2 inhibitor use across the first six NDR registrations.

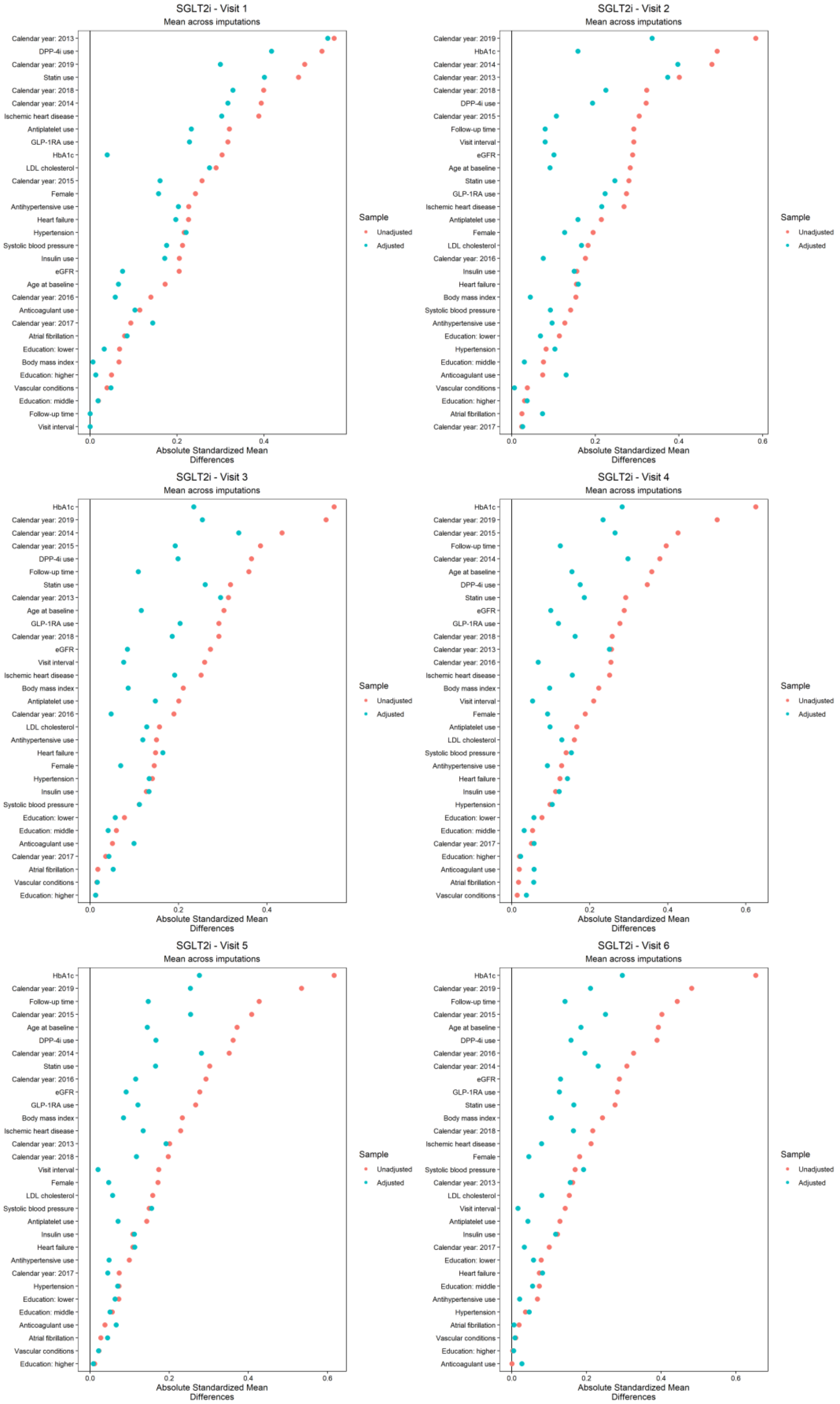

Supplementary Figure 5. Standardized mean differences before and after inverse probability of treatment weighting for GLP-1 receptor agonist use across the first six NDR registrations.

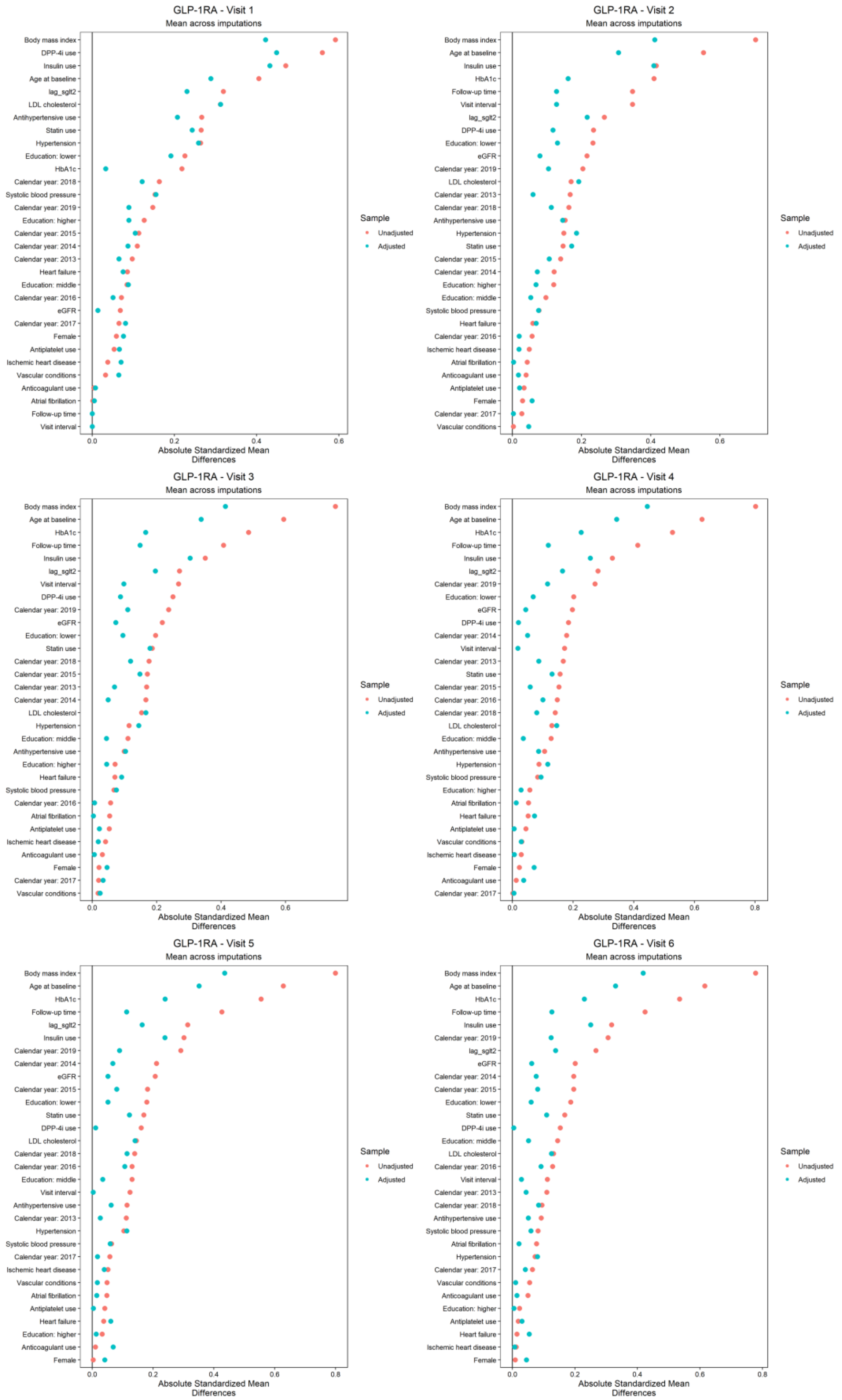

Supplementary Figure 6. Standardized mean differences before and after inverse probability of treatment weighting for DPP-4 inhibitor use across the first six NDR registrations.

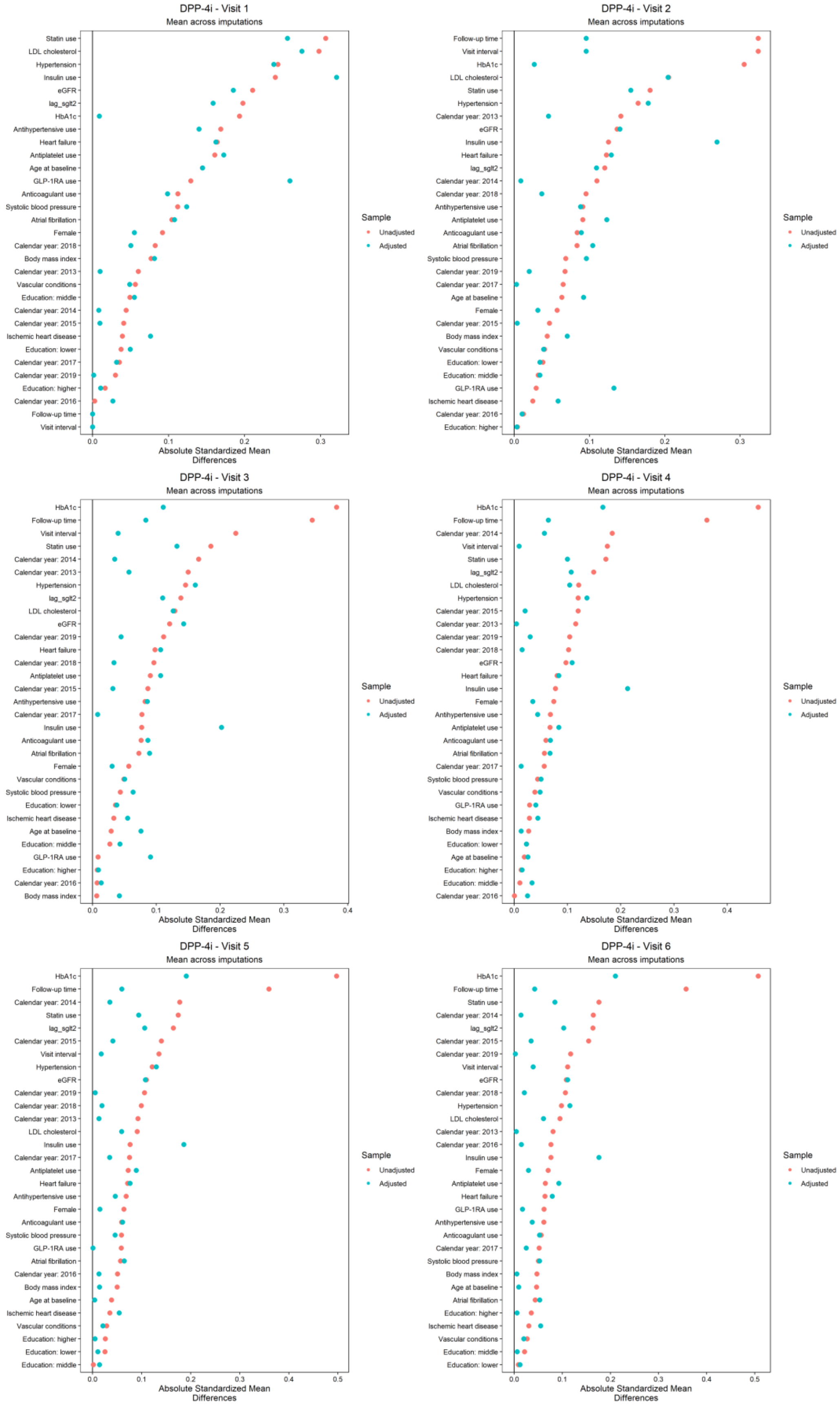

Supplement: Supplementary file 1 [file mmc1.pdf]
